# Analysis of optical coherence tomography biomarker probability detection in central serous chorioretinopathy by using an artificial intelligence-based biomarker detector

**DOI:** 10.1186/s40942-024-00560-6

**Published:** 2024-05-31

**Authors:** Lorenzo Ferro Desideri, Rodrigo Anguita, Lieselotte E. Berger, Helena M. A. Feenstra, Davide Scandella, Raphael Sznitman, Camiel J. F. Boon, Elon H. C. van Dijk, Martin S. Zinkernagel

**Affiliations:** 1 Department of Ophthalmology, Inselspital, Bern University Hospital, University of Bern, Freiburgstrasse 15, Bern, CH-3010 Switzerland; 2 Department for Bio Medical Research, University of Bern, Murtenstrasse 24, Bern, CH-3008 Switzerland; 3 Bern Photographic Reading Center, Inselspital, University Hospital Bern, Bern, 3010 Switzerland; 4 Department of Ophthalmology, Leiden University Medical Center, Leiden, the Netherlands; 5 ARTORG Research Center Biomedical Engineering Research, University of Bern, Bern, Switzerland; 6 Department of Ophthalmology, Amsterdam University Medical Centers, Amsterdam, the Netherlands; 7 Moorfields Eye Hospital, NHS Foundation Trust, London, UK

## Abstract

**Aim:**

To adopt a novel artificial intelligence (AI) optical coherence tomography (OCT)-based program to identify the presence of biomarkers associated with central serous chorioretinopathy (CSC) and whether these can differentiate between acute and chronic central serous chorioretinopathy (aCSC and cCSC).

**Methods:**

Multicenter, observational study with a retrospective design enrolling treatment-naïve patients with aCSC and cCSC. The diagnosis of aCSC and cCSC was established with multimodal imaging and for the current study subsequent follow-up visits were also considered. Baseline OCTs were analyzed by an AI-based platform (Discovery® OCT Fluid and Biomarker Detector, RetinAI AG, Switzerland). This software allows to detect several different biomarkers in each single OCT scan, including subretinal fluid (SRF), intraretinal fluid (IRF), hyperreflective foci (HF) and flat irregular pigment epithelium detachment (FIPED). The presence of SRF was considered as a necessary inclusion criterion for performing biomarker analysis and OCT slabs without SRF presence were excluded from the analysis.

**Results:**

Overall, 160 eyes of 144 patients with CSC were enrolled, out of which 100 (62.5%) eyes were diagnosed with cCSC and 60 eyes (34.5%) with aCSC. In the OCT slabs showing presence of SRF the presence of biomarkers was found to be clinically relevant (> 50%) for HF and FIPED in aCSC and cCSC. HF had an average percentage of 81% (± 20) in the cCSC group and 81% (± 15) in the aCSC group (*p* = 0.4295) and FIPED had a mean percentage of 88% (± 18) in cCSC vs. 89% (± 15) in the aCSC (*p* = 0.3197).

**Conclusion:**

We demonstrate that HF and FIPED are OCT biomarkers positively associated with CSC when present at baseline. While both HF and FIPED biomarkers could aid in CSC diagnosis, they could not distinguish between aCSC and cCSC at the first visit. AI-assisted biomarker detection shows promise for reducing invasive imaging needs, but further validation through longitudinal studies is needed.

## Introduction

Central serous chorioretinopathy (CSC) has been classically subdivided into acute (aCSC) and chronic (cCSC) subtypes [1]. While aCSC typically resolves spontaneously within 3 to 6 months, cCSC is characterized by persistence of subretinal fluid (SRF) longer than 4 to 6 months. Furthermore, fluorescein angiography (FA) classically shows the presence of focal leakage in acute forms, whereas this leakage usually appears diffuse or multifocal in cCSC [2]. Despite this background, the clinical manifestations of CSC and the course of disease may be heterogenous in everyday practice and there is still poor consensus about CSC classification among retina specialists. In fact, the current classification is merely based on a temporal criterion, which is arbitrarily set between 3 and 6 months, and the clinical phenotypes may overlap in some cases [3, 4]. To improve the present CSC classification, Chhablani et *al.* recently suggested dividing ‘simple CSC’ from ‘complex CSC’, by evaluating the extent of retinal pigment epithelium (RPE) alterations [5]. In this direction, another recent study showed the presence of different genetic and clinical features comparing simple CSC and complex CSC [6]; however, further evidence is needed to validate this proposed classification.

With the aim of allowing a better CSC classification, the analysis of optical coherence tomography (OCT) biomarkers has been recently performed in different studies to better characterize and distinguish the 2 different clinical subtypes [7, 8]. Although some OCT biomarkers have been related to spontaneous resolution of CSC, including the extent of ellipsoid zone abnormalities, SRF height, central macular thickness, and subfoveal choroidal thickness, to date no consistent evidence has been provided yet in terms of OCT imaging features typical for the disease phenotype [8]. Artificial intelligence (AI) applied to the analysis of fundus and OCT images has recently shown promising results in predicting disease recurrence in CSC [9, 10].

In our study, we performed a comprehensive analysis of the OCT biomarkers by adopting an AI-OCT based fluid biomarker detector. Our aim was to identify what OCT biomarkers were correlated to a specific disease phenotype, namely aCSC or cCSC.

## Methods

### Study subjects and design

We performed a multicenter, retrospective, comparative clinical series on CSC patients who had a follow-up of at least 1 year. Based on the baseline visit and the clinical course and multimodal imaging at that subsequent visit, the distinction between aCSC and cCSC could be made. This study was carried out at the Department of Ophthalmology at Inselspital, Bern University Hospital, (Bern, Switzerland) and at the Department of Ophthalmology at Leiden University Medical Center (Leiden, the Netherlands). Informed consent was acquired from all patients and institutional review board approval was obtained from both centers and all aspects of this retrospective study adhered to the tenets of the Declaration of Helsinki.

The diagnosis of CSC was based on clinical history, clinical examination, and multimodal imaging, including OCT, fundus autofluorescence (FAF), FA, and indocyanine angiography (ICGA). Retrospectively, CSC patients in whom SRF spontaneously resolved within 4–6 months from the onset of symptoms were included in the aCSC group, whereas patients who showed persistence of SRF after 6 months were diagnosed with cCSC.

Distinction between aCSC and cCSC was also supported by multimodal imaging. Patients in the cCSC group needed to present with multifocal areas of RPE alteration on FAF as well as multiple RPE leaks at FA. By contrast, patients in the aCSC group typically had a single fluorescein leakage site and no other FA anomalies. Both in aCSC and cCSC, hyperfluorescent changes with an indistinct border – typical of diseases that are part of the pachychoroid disease spectrum – had to be present. Exclusion criteria were the presence of other macular diseases, prior PDT or other types of macular laser treatment, other interventions such as anti-VEGF injections, prior history of vitreoretinal surgery, uveitis, glaucoma, optic nerve disease, high myopia of more than …diopters and a positive family history of systemic diseases that could affect the macular status. Patients presenting at baseline with advanced clinical signs of chronic damage at the level of the RPE on the OCT and FAF (hypo/hyperreflective and descending tract AF patterns [11]) were also excluded.

### OCT imaging

OCT volumes were acquired from CSC patients from the databases of the 2 participating hospitals (University Hospital Inselspital and Leiden University Medical Center). The Spectralis SD-OCT imaging system was used (Heidelberg Engineering Inc., Heidelberg, Germany). OCT volumes covering an area of 5.90 mm × 5.75 mm × 1.92 mm centered on the fovea with a 49-B scan acquisition protocol and a resolution of 496 × 512 pixels per B-scan were examined. For each horizontal scan, 49 B-scans were averaged. Patients with poor image quality and/or artifacts were ruled out from the analysis. For both patients in the aCSC and cCSC groups, only baseline OCT images were analyzed.

### Quantitative analysis: AI-based biomarker OCT detector

**Fig. 1:**
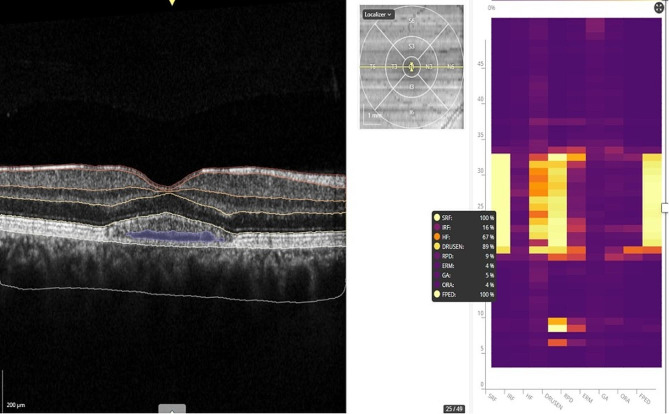
Percentage of biomarkers automatically detected by Discovery® for each OCT-b scan slice. FIPED = flat irregular pigment retinal epithelium detachment; HF = hyperreflective foci; IRF = intraretinal fluid; SRF = subretinal fluid

To perform OCT images analysis, we used the AI-based platform Discovery® OCT Biomarker Detector (RetinAI AG, Switzerland) [12]. This software automatically computes the probability of presence (a number ranging from 0 to 100%) of the following biomarkers: subretinal fluid (SRF), intraretinal fluid (IRF), hyperreflective foci (HF) and flat irregular pigment epithelium detachment (FIPED) The definitions of each single OCT biomarker are listed in the table (Table 1) The output format of the biomarker detection is a list of probabilities with one value per biomarker per B-scan, as shown in the figure **(**Fig. 1**)**. The OCT biomarkers were quantified in the macular region in each B-scans where SRF was present, and an average value was therefore calculated. The presence of SRF was considered as a necessary inclusion criterion for performing biomarker analysis and OCT slabs without SRF presence were excluded from the analysis. Average probability values higher than 50% were arbitrarily considered to be clinically relevant according to the ‘precision and recall’ principle for the presence of a certain imaging biomarker. This concept was previously explained in the study by Kurmann et *al.* [13]. Next, the average measurements at the first visit were compared between patients in the aCSC and cCSC groups to describe the differences in the percentage of OCT biomarkers present at baseline between the two groups.

### Statistical analysis

Statistical analysis was performed by using Python (3.1) statistics software. The normality of the data was tested by using the Shapiro-Wilk test. The difference between sample means of biomarkers percentage in aCSC and cCSC groups was tested for significance by means of a Welch’s t-test. A Bonferroni correction was done to adjust for multiple testing. With 4 measured biomarkers, a p value of α < 0.05/4 (0.0125) was considered statistically significant.

## Results

### Demographic and clinical features

We included 160 eyes of 144 patients with CSC, out of which 100 eyes (62.5%) were diagnosed with cCSC and 60 eyes (34.5%) with aCSC. In the aCSC group, the mean age was 48.0 years (± 11.2 years), whereas in the cCSC group this was 55.7 years (± 10.4 years), showing a statically significant difference between the 2 groups (*p* = 0.001). In the aCSC group, 77.6% of patients were male compared to 79.0% in the cCSC group (*p* = 0.705). The average spherical equivalent (SE) at baseline was + 0.56 D (± 1.58 D) in the aCSC group, while it was + 0.74 D (± 3.1 D) in the cCSC group (*p* = 0.144). Furthermore, steroid use was reported in 26.6% (16/60) of the patients in aCSC group as opposed to 17.0% (17/100) in the cCSC group, (*p* = 0.163). Baseline characteristics of the patients included in the current study are summarized in the table **(**Table 2**)**.

**Table 1:** OCT biomarkers definition allowed by discovery

Biomarker	Definition
SRF	Well-defined darkening with a minimal horizontal extension of 100 μm between the retinal pigment epithelium layer (RPE) and photoreceptor layer
IRF	Diffuse darkening and thickening of the neurosensory retina and oval well-defined hyporeflective areas with a minimal extension of 25 μm in any direction between the internal limited membrane and the photoreceptor layer
HF	Small points of increased reflectivity scattered throughout all retinal layers, primarily found in near vicinity of intraretinal cystoid spaces. The size of HF can vary from 25 μm in diameter to 50 μm and they can be clustered
FIPED	Detachment of the RPE from Bruch’s membrane characterized by a hyperreflective structure underneath the RPE. Often the fibrovascular PED presents as an undulating RPE and a “low-lying” PED, referred to as “double layer sign’

FIPED = flat irregular pigment retinal epithelium detachment; HF = hyperreflective foci; IRF = intraretinal fluid; SRF = subretinal fluid [13]

**Table 2:** Baseline characteristics of patients with central serous chorioretinopathy included in the current study

Demographic and/or clinical feature	aCSC *n* = 60	cCSC *n* = 100	*p* value
Age, years, mean ± SD	48.0 ± 11.2	55.7 ± 10.4	**< 0.001**
Sex, *n* (%)			
Male	46 (77.6)	79 (79.0)	0.705
Female	14 (22.4)	21 (21.0)	
Laterality, *n* (%)			
Right	33 (55.0)	49 (49.0)	0.737
Left	27 (45.0)	51 (51.0)	
Ethnicity, *n* (%)			
Caucasian	55 (91.7)	85 (85.0)	
Asian	0 (0)	2 (2.0)	
Hispanic	2 (3.3)	1 (1.0)	0.225
Middle East-Arabic	3 (5.0)	12 (12.0)	
Baseline SE, D, mean ± SD	+ 0.56 D ± 1.58	+ 0.74 D ± 3.1	0.144
Steroid use *n* (%)	16 (26.6)	17 (17.0)	0.163

aCSC = acute central serous chorioretinopathy; cCSC = chronic central serous chorioretinopathy, SD = standard deviation, SE = standard error

### Analysis of biomarkers

Our analysis revealed that in the OCT slabs showing presence of SRF the average percentage of biomarkers was found to be > 50% for HF and FIPED in both the CSC groups. This contrasts with IRF showing mean percentages < 50% both in patients with aCSC and cCSC.

**Fig. 2:**
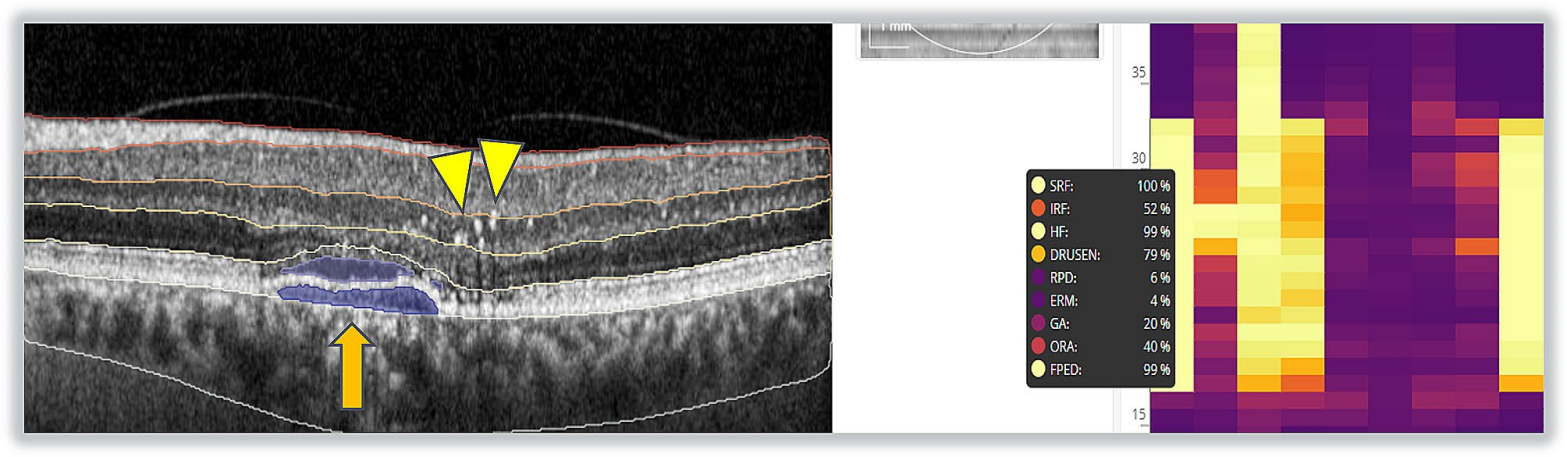
OCT slab of a patient with chronic central serous chorioretinopathy the clinically relevant biomarkers associated with the presence of the disease can be detected with high reliability by the software (probability of 99%). The orange arrow highlights the presence of a distinct flat irregular pigment epithelium detachment, whereas the yellow arrow heads indicate the presence of several hyperreflective foci

Subgroup analysis showed that HF were found in 81% (± 20) of eyes in the cCSC group and 81% (± 15) of eyes in the aCSC group (*p* = 0.4295). FIPED had a mean overall percentage of 88% (± 18) in cCSC and 89% (± 15) in aCSC (*p* = 0.3197). The probability to find IRF was on average 35% (± 18) in patients in the aCSC group in comparison with on average 28% (± 14) in patients in the cCSC group (*p* = 0.0095) **(**Table 3) (Fig. 2).

**Table 3:** Mean percentage of OCT biomarkers at first visit in patients with acute vs. chronic central serous chorioretinopathy

Retinal biomarker	aCSC (mean percentage ± SD)	cCSC (mean percentage ± SD)	*p* value
FIPED	88 ± 18	89 ± 15	0.3197
HF	81 ± 20	81 ± 15	0.4295
IRF	28 ± 14	35 ± 18	**0.0095**

aCSC = acute central serous chorioretinopathy; cCSC = chronic central serous chorioretinopathy; FIPED = Fibrovascular Retinal Pigment Epithelium Detachment; HF = Hyperreflective Foci

## Discussion

In this retrospective, multicenter study we used an AI-based program to investigate OCT biomarkers in patients with CSC and whether these could be used to differentiate between aCSC and cCSC. We observed that our program was able to detect the presence of HF and FIPED at clinically meaningful percentages in our cohort of CSC patients, suggesting that they could be adopted as possible biomarkers to indicate the presence of disease. These biomarkers were identified at baseline examination for both CSC forms in the OCT scans with SRF. However, no differences for HF and FIPED were found between aCSC and cCSC.

Previous studies have utilized specific OCT parameters, like increased choroidal thickness (CT), elevated choroidal vascularity index (CVI) and choroidal HF to identify and diagnose CSC [7, 8, 14, 15]. However, no consistent evidence for distinctive OCT biomarkers to establish the CSC diagnosis? have been found yet. This study represents the first attempt to quantitatively analyze the likelihood of finding OCT biomarkers specifically related to CSC, with a particular focus on AI-automatically detected biomarkers: HF, FIPED and IRF.

HF have been associated with several retinal diseases including CSC [16]; they are usually located in the outer retina, in the subretinal space, and sub-RPE area and correspond with the leakage site identified by FA. A previous study using high-resolution retinal imaging in patients with CSC showed the presence of HF in the outer retinal and subretinal layers [17]. They found that these yellowish dot-like precipitates are not only confined to the posterior surface of the detached retina but are also present within the detached neurosensory retina. The authors proposed that these intraretinal precipitates could have originated from the accumulation of proteins or macrophages containing phagocytized photoreceptor outer segments [17]. Lee at *al.* reported that the baseline presence of retinal HF was a predictor of anatomical and functional recovery and a higher number of HF was associated with recurrence tendency in patients with CSC [18]. In another study, the presence of HF in the choroid was studied in relation to aCSC and cCSC. It was revealed that choroidal HF were significantly correlated with the remodeling of chorioretinal structures, and they were found to be present in a greater amount in aCSC in comparison with cCSC. The authors hypothesized that retinal HF primarily resulted from microglial cell activation, which was associated with the phagocytosis of photoreceptor outer segments. In contrast, the presence of HF in the choroid might be attributed to a combination of inflammatory processes and vascular changes occurring within the choroidal vasculature [19].

In our study, we found that the baseline presence of retinal HF was clinically meaningful for CSC diagnosis; however, discerning between aCSC and cCSC was not possible. These findings seem to suggest that AI-assisted quantification of retinal HF may be indicative for diagnosis confirmation but cannot help in distinguishing the clinical course of CSC. In contrast to the study of Hanumunthadu et *al.* [19], which found more choroidal HF in the aCSC group, the divergent results might stem from discrepancies in the anatomical origin of HF (retina vs. choroid).

Further studies based on AI-programs should better clarify if retinal and choroidal HF may have a predictive role as well as a diagnostic role in CSC.

We also found that FIPED, described as a shallow and irregular elevation of RPE from Bruch’s membrane, was a clinically meaningful biomarker indicative of CSC presence. This clinical sign has been classically associated with chronic forms of CSC, in contrast with the more typical dome-shaped PED observed in patients with aCSC [20]. Previous studies have reported that most of the FIPEDs are avascular and only 18.9% of them harbor the presence of an underlying neovascularization demonstrated by FA [21]. A previous study described the different OCT features between avascular and vascular FIPEDs, including a lower subfoveal CT and increased CVI in vascular FIPEDs [22]. In our study, we only analyzed avascular FIPEDs, since we examined only baseline OCT visits and no patient in our cohort showed signs of neovascularization on multimodal imaging at the first visit. A recent longitudinal study showed that FIPEDs were positively associated with cCSC rather than aCSC, in absence of underlying neovascular processes [23].

Consistent with prior research, our study demonstrated an elevated occurrence of FIPEDs in both aCSC and cCSC groups during the initial examination. This suggests that FIPED could serve as a valuable biomarker indicating the presence of the disease at baseline; however, based on our findings, FIPED cannot be used to differentiate between the clinical course of CSC if measured at the first visit. To gain more clarity on this matter, additional longitudinal studies with well-structured follow-up periods should assess the differences in FIPED percentages between patients with aCSC and cCSC.

Lastly, we found statistically significant differences between aCSC and cCSC patients in terms of IRF; however, the relatively low mean probabilities (all of them lower on average than 50%) associated with these biomarkers do not suggest us to consider their presence clinically relevant and significant to draw consistent conclusions on. In this regard, we deem that further validation with Discovery® is needed to better define the critical threshold for the presence of a certain biomarker. However, it is highly improbable for OCT scans to reveal the presence of that specific biomarker when percentages are below 50% [12].

In conclusion, our study presents the first AI-based program to detect OCT biomarkers associated with CSC. HF and FIPED biomarkers were found to be clinically meaningful indicators of CSC. While both HF and FIPED could aid in CSC diagnosis, they could not distinguish between aCSC and cCSC when analyzing the first OCT visit. The potential of these biomarkers could reduce the need for invasive imaging techniques and improve patient comfort during diagnosis. Further longitudinal studies are required to validate these findings and determine critical thresholds for biomarker presence. The adoption of AI-assisted biomarker detection has promising implications for routine clinical practice.

## Data Availability

All data are available and kept in Inselspital protected database.
